# Phylogenetic Analysis and Characterization of the Complete Hepatitis E Virus Genome (Zoonotic Genotype 3) in Swine Samples from Mexico

**DOI:** 10.3390/v10080391

**Published:** 2018-07-26

**Authors:** Alicia Sotomayor-González, María E. Trujillo-Ortega, Blanca I. Taboada-Ramírez, Carlos Sandoval-Jaime, Rosa E. Sarmiento-Silva

**Affiliations:** 1 Laboratory of Virology, Microbiology and Immunology Department, Veterinary Medicine and Husbandry Faculty, National Autonomous University of Mexico (UNAM), Mexico City 04510, Mexico; mvzaliciasotomayor@gmail.com; 2 Academic Council of the Biological, Chemical and Medical Sciences, National Autonomous University of Mexico (UNAM), Mexico City 04510, Mexico; elenam@unam.mx; 3 Swine Medicine and Husbandry Department, Veterinary Medicine and Husbandry Faculty, National Autonomous University of Mexico (UNAM), Mexico City 04510, Mexico; 4 Biotechnology Institute (IBT), National Autonomous University of Mexico (UNAM), Cuernavaca 62209, Morelos, Mexico; btaboada@ibt.unam.mx (B.I.T.-R.); carlossj@ibt.unam.mx (C.S.-J.)

**Keywords:** hepatitis E, hepatitis, swine, zoonosis, bile, characterization

## Abstract

Hepatitis E virus (HEV) is an emerging public health problem with an estimated 20 million infections each year. In Mexico, *Orthohepevirus* A, genotype 2, has been reported in humans, but genotype 3 has only been reported in swine (zoonotic). No diagnostic tests are publicly available in Mexico, and only partial sequences have been reported from swine samples. Hence, research is necessary to determine circulating strains, understand the features and dynamics of infection on pig farms, determine how to implement surveillance programs, and to assess public health risks. In this study, a next-generation sequencing (NGS) approach was applied to obtain a complete genome of swine HEV. Liver, feces, and bile samples were taken at slaughterhouses and a farm in Mexico. RT-PCR was used to determine positive samples and confirmed by Sanger sequencing. Of the 64 slaughterhouse samples, one bile sample was positive (B1r) (1.56%). Of 21 sample pools from farm animals, 14 were positive (66.66%), representing all stages of production. A complete sequence strain MXCDg3_B1c|_2016 was obtained from the bile of a domestic swine in the fattening stage. In addition, two partial sequences—MXCDg3_H2cons|_2016 (1473 nt) and MXCDg3_C3Acons|_2016 (4777 nt)—were obtained from sampled farm animals. Comparison with all reported genome HEV sequences showed similarity to genotype 3 subgenotype a (G3a), which has been previously reported in acute cases of human hepatitis in the US, Colombia, China, and Japan.

## 1. Introduction

Viral hepatitis is considered as one of the main causes of hepatic disease in humans worldwide. Five known viruses have been associated with hepatitis: hepatitis A virus, hepatitis B virus, hepatitis C virus, hepatitis D virus, and hepatitis E virus. However, of these, only hepatitis E has been reported as a zoonotic disease. Hepatitis E is an emerging public health problem, and in many countries the disease is known to cause epidemics [1,2]. The World Health Organization (WHO) website reports that there are an estimated 20 million hepatitis E infections each year of which 3 million have an acute presentation resulting in up to 56,600 deaths [3].

Hepatitis E virus (HEV) capsid is 27–34 nm in diameter with icosahedral symmetry and a single-stranded, positive-sense RNA genome of 7.2 kb. The viral genome contains short non-coding regions (IRES) of 27–35 nucleotides (nt) at the 5’ end and 65–75 nt in the 3’ region, with a poly A tail. It contains three discontinuous, partially superimposed open reading frames (ORF1–3) [4,5]. The large ORF1 encodes the non-structural proteins, which contain conserved peptide domains essential for viral replication and consist mainly of methyltransferase, protease, macrodomain, helicase, and an RNA-dependent RNA polymerase (RdRp). It also encodes for the X, Y, and V (variable) domains of unknown functions [6]. ORF2 and ORF3 overlap and are translated from a single bicistronic subgenomic RNA that is 2.2 kb in length [7]. ORF2 encodes the structural capsid for which three different forms have been reported—infectious/intracellular (ORF2i), glycosylated (ORF2g), and cleaved (ORF2c) [8,9,10]. The protein has three defined domains: S (shell), M (middle), and P (protruding) composed of amino acid residues 118–313, 314–453, and 454–606, respectively. ORF3 encodes a 113 or 114 aa phosphoprotein that is involved in assembling and releasing viruses [1,11].

HEV is classified in the genus *Orthohepevirus*, which is divided into four species as follows: *Orthohepevirus* B viruses affect birds, *Orthohepevirus* C viruses affect small mammals and rodents, *Orthohepevirus* D virus has only been found in bats, and *Orthohepevirus* A viruses have been found in various mammals, including deer, camels, rabbits, wild boars, swine, and humans. Swine are the principle reservoirs of the disease, and zoonotic transmission has been reported extensively. Specifically, transmission has been reported through consumption of uncooked pork products, with cases of grilled meat from a wild boar in Japan [12], raw pig liver sausages in France [13], and pork meat in Spain [14] directly being associated with the disease by genotyping of the patient sample and the food sample. Likewise, higher seroprevalence values have been found in people who work in close association with swine, such as farmworkers and slaughterhouse personnel [15,16,17,18]. In Bangladesh, 28% of pig handlers reported jaundice in a two-year period, whereas only 17% of people with no contact with swine reported jaundice [15]; swine veterinarians in the US were 1.51 times more likely to present HEV antibodies against swine HEV antigen [16], while swine farmers in Taiwan reported approximately 3.5-fold increased risk [17]. The presence of the virus in farms might also implicate a risk for environmental transmission, with contaminated water being associated with outbreaks [19,20,21].

Eight different genotypes have been identified for *Orthohepevirus* A. In Mexico, genotype 2 has been reported in humans and is commonly referred to as the “Mexican strain” [22,23]. Genotype 3—a zoonotic strain—has been reported in Mexico but exclusively in swine. Several studies of HEV have been conducted in pigs but recent work have only succeeded in detecting antibodies against the virus using serological tests [24,25]. Furthermore, genotype 3 infections have been emerging recently in America, likely due to contaminated blood transfusions and consumption of contaminated food [8].

Regarding pig populations in Mexico, information on the presence of the virus is still scarce. Little importance has been given to the disease because of the lack of clinical signs affecting swine and the deficiency of the diagnostic tests available for pigs and humans. However, Panduro et al. reported a 12% rate of clinical viral hepatitis in humans where no etiological agent is diagnosed [26]. No publicly available diagnostic tests are performed for HEV in Mexico, so it could be involved in a high proportion of these cases. Thus, better knowledge of circulating strains, especially zoonotic ones, and their relation to strains around the world and from different hosts might provide a broader picture of the disease in Mexico and help develop control measures for at-risk populations.

This study determined the full-length genome sequence of HEV strain MXCDg3_B1c|_2016 and of two partial sequences that comprise a large part of the genomes—MXCDg3_H2cons|_2016 (2464–7241 nt) and MXCDg3_C3Acons|_2016 (5766–7239 nt)—isolated from samples of domestic swine (Sus scrofa) taken at different production stages. Comparison of the sequences obtained with those available in GenBank databases showed that they are similar to the genotype 3 subgenotype a (G3a). The presence of the zoonotic genotype in feces on farms indicates the need for stricter biosecurity measures to prevent transmission of the disease to workers and possible dissemination to the environment. The incidence of this disease in Mexico is likely underestimated due to the lack of available diagnostic techniques in the country and the lack of apparent damage in pigs that would affect the productive parameters by itself.

## 2. Materials and Methods

### 2.1. Sample Collection

To determine the presence or absence of the virus, the Cannon and Roe formula (1982) was applied at a confidence level of 95% and an error of 5% using a previously reported prevalence of 19%; this resulted in a minimum of 16 samples [27,28]. Two sampling strategies were followed. First, samples were collected at 4 slaughterhouses: 1 in the State of Mexico and 3 in Veracruz. Samples of liver, bile, and feces were gathered from 16 individuals at each site. Second, sampling was conducted at a farrowing-to-finishing farm, where 30 fecal swabs from different production stages (weaning, postweaning, finishing, replacement sows, and boars) were taken, and necropsies were performed on 8 animals in the fattening stage.

### 2.2. Molecular Detection of HEV

RNA extraction was carried out following the phenol technique [29]. Reverse transcriptase was performed using SuperScript™ II Reverse Transcriptase (Invitrogen, Carlsbad, CA, USA) following the manufacturer’s instructions. Platinum Taq DNA Polymerase (Invitrogen) was used for PCR following the instructions for the kit and primers and conditions from de Deus and/or Huang [30,31]. RT-PCR positive control was an amplification product of 216 bp, kindly donated by Dr Marga Martin at the Autonomous University of Barcelona [21]. PCR amplicons were visualized in 2% agarose gel. Positive samples were purified with E-Gel size select II Agarose Gels at 2% (Thermo Fisher, Waltham, MA, USA) and sent to the Biomedical Research Institute (IIB) for Sanger sequencing to confirm the presence of hepatitis E virus.

### 2.3. HEV Sequencing

RNA concentrations were measured (Genova Nano micro-volume spectrophotometer 737,501, Jenway, Staffordshire, UK) in the 15 positive samples after phenol extraction, and the samples with the highest concentrations were selected for a directed metagenomic analysis. Five samples were taken to the Sequencing Facility at the Biotechnology Institute (IBT, Cuernavaca, Morelos, Mexico). Pretreatment with PoliT pearls (pearls with oligo poly dT, TruSeq Stranded mRNA Library Prep Kit by Illumina, San Diego, CA, USA) was performed on all samples to direct target sequencing to poliA tail sequences. RNA was sent to the Biotechonolgy Institute (IBT) where next-generation sequencing was performed with NEXTSeq 500, ILUMINA, equipment.

### 2.4. Computer Sequence Analysis

For each sample, a preprocess analysis was performed with cutadapt (1.11) to remove adaptors and smaller reads. After that, duplicated reads were eliminated with Cd-Hit_dup with a 100% value (1 × 10^−3^) [32], and unique reads were mapped at 90% identity with BBMap v36.99 against an HEV reference genome (GenBank AF060669.1), eliminating regions with quality below 15 [33]. The parameters used for BBMap were trimq = 15, minid = 0.9, sam = 1.3. Next, hit-reads were used to obtain a consensus sequence using Samtool 1.3.1 and bcftools (1.3.1) with manual verification to correct possible errors [34,35]. Depth coverage and map quality were assessed using Qualimap 2.2.1 [36].

NCBI BLAST (2.8.5.0) (https://blast.ncbi.nlm.nih.gov/Blast.cgi) was used to verify our genome with a standard nucleotide blast to ensure that the sequence obtained matched only HEV virus sequences [37]. Finally, protein annotation was performed with the ExPASy translation tool (www.expasy.org) for frame detection of the three open reading frames.

### 2.5. Phylogenetic Analysis

All 154 complete genome sequences of HEV available in GenBank as of January 2018 for genotype 3 were downloaded as well as at least one reference sequence for genotypes 1, 2, and 4–7. CD-Hit 4.6.8 was used to eliminate sequences with >98% similarity (−c.98), leaving a total of 100 reference sequences. Following this, alignment of the consensus and reference sequences was performed with Clustal W (default parameters) and verified manually using Mega7 [38].

Phylogenetic analysis of the complete genome and also to capsid protein analysis were inferred by the maximum likelihood method based on the GTR model with a discrete Gamma distribution, which was selected as the best fit model, and 1000 bootstrap value. This analysis involved 101 nucleotide complete sequences, 6299 nucleotides long, and 102 nucleotide capsid sequences, 1861 nucleotides long. These sequences are identified by country (first two letters), host (HM = human, CD = swine, DE = deer), genotype (g1–7), subgenotype if reported (a–k), GenBank accession number, and year of collection.

## 3. Results

### 3.1. Molecular Detection of Hepatitis E

Of the 64 individuals sampled at the four slaughterhouses, only one bile sample proved to be positive (B1) (1.56%). By contrast, of the 21 sample pools processed from the farm, 14 were positive (66.66%) and represented all different stages of production (Table 1).

### 3.2. HEV Sequencing

From the five samples selected for next-generation sequencing, we obtained 522,055 reads for sample B1, 623,888 for sample C3A, 301,739 for sample C5A, 694,653 for sample H2, and 1,290,744 for sample H3. The reads obtained had an average length of 75 base pairs (bp). After preprocessing and CD-Hit analysis, 315,714 sequences remained for B1, 401,402 for C3A, 96,649 for C5A, 443,479 for H2, and 180,728 for H3.

**Figure 1:**
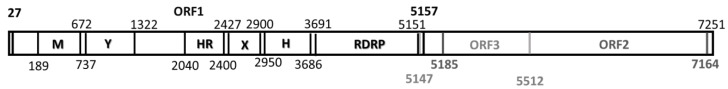
Graphic representation of the complete sequence MXCDg3_B1c|_2016 and capsid analysis. Complete sequence of 7241 nucleotides consisting of 5’ UTR of 27 nt (1–27), ORF1 of 5120 nt (27–5147), ORF2 of 1979 nt (5185–7164), ORF3 of 365 nt (5147–5512), and a 3’ UTR of 77 nt (7164–7251).

Hepatitis E virus reads were obtained from only three samples, as follows:

(i) Bile sample B1. We were able to assemble one complete hepatitis E virus genome, denoted MXCDg3a_B1c|_2016 with a coverage of 354.62. This genome is 7241 nucleotides (nt) long, excluding the poli-A tail (12 nt) at the 3’ termini. The genome consisted of 5’ UTR of 27 nt (1–27); three open reading frames—ORF1 of 5120 nt (27–5147), ORF2 of 1979 nt (5185–7164), ORF3 of 365 nt (5147–5512)—and a 3’ UTR of 77 nt (7164–7241), followed by the poli-A tail (Figure 1). This sequence had been deposited at GenBank under accession no. MG833836.
(ii) Liver sample H2 was denoted MXCDg3a_H2cons|_2016; a partial sequence was obtained of 1473 nt covering the 3’ end (5766–7239) with a coverage of 6.30. The GenBank accession number is MH003296.
(iii) Feces sample C3A was identified as MXCDg3a_C3Acons|_2016; a partial sequence of 4777 nt was obtained covering the 3’ end (2464–7241) with a coverage of 18.35; GenBank no. MG980615.

No HEV sequences were obtained from samples H3 and C5A.

### 3.3. Complete Genome Characterization

The complete genome MXCDg3_B1c|_2016 was analyzed using BLASTN to identify sequence homology with reported hepatitis E virus sequences. To characterize the genome, a BLASTN between the references obtained was performed. The complete genome sequence MXCDg3_B1c|_2016 shared a high nucleotide identity with reference sequences of genotype 3 (Table 2). The highest nucleotide identity of 91% was shown with sequences isolated from humans and pigs in the US, Canada, Japan, and the United Kingdom. The ORF1, ORF2, and ORF3 identities were 91%, 92%, and 98% for nucleotides, and 98%, 99%, and 98% at amino acid level, respectively. The nucleotide identities of MXCDg3a_B1c|_2016 with genotype 1, genotype 2 (Mexican strain), and genotype 4 were 77%, 77%, and 78%, respectively.

### 3.4. Phylogenetic Analysis

**Figure 2:**
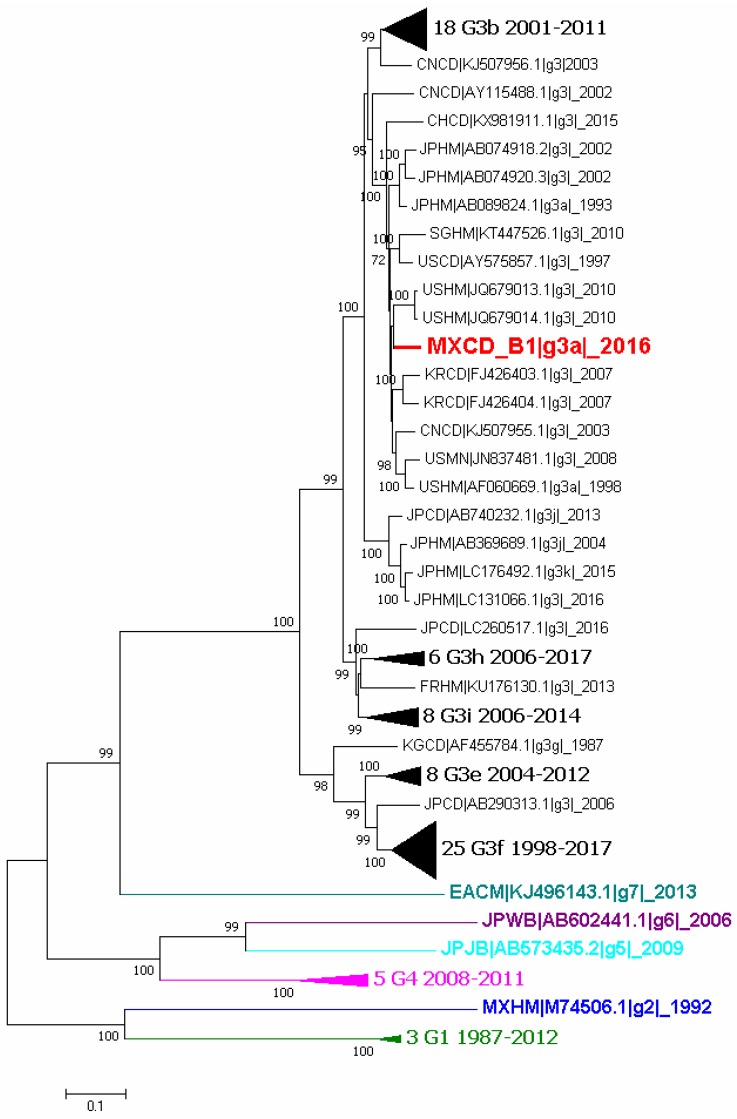
Phylogenetic tree of all available complete genome sequences of hepatitis E virus (HEV). Analysis included 101 nucleotide sequences (CD-HIT 98%). Relationship was inferred by the maximum likelihood method based on the GTR model with gamma distribution and 1000 bootstrap. Branches were clustered to construct the graph. The first number indicates the number of sequences, genotype and subgenotype, and year of detection. Black triangles symbolize clustering of similar sequences identified by number of sequences, genotype and years of report. Colors represent different genotypes, blue for genotype 2, green for genotype 3, magenta for genotype 4, cyan for genotype 5, purple for genotype 6 and light blue for genotype 7.

Phylogenetic analyses were performed using complete genome sequences and the capsid ones. The first analysis was constructed with sequences of genotype 3 reported in GenBank and also strains of other genotypes. The complete B1 sequence was included (MXCDg3_B1c|_2016) in the analysis and, as shows Figure 2, it clusters with genotype 3 subgenotype a (G3a).

**Figure 3:**
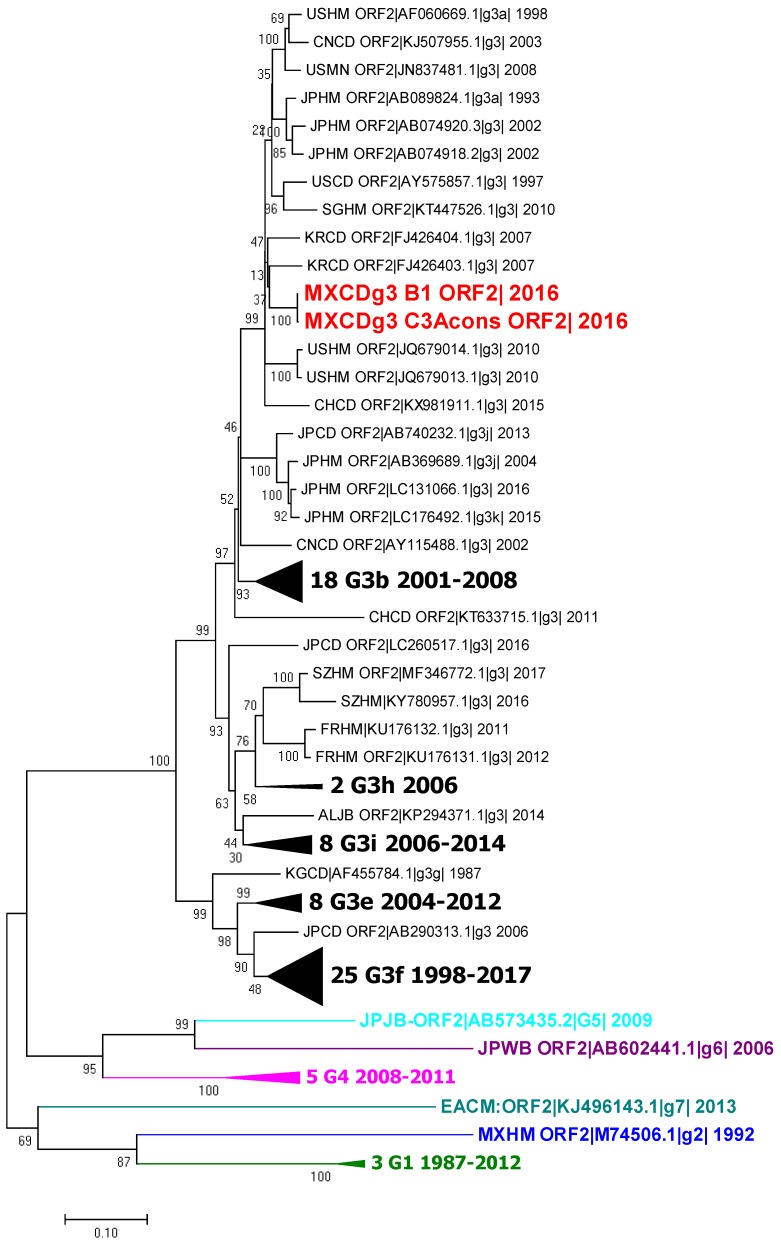
Phylogenetic tree of the ORF2 sequences of HEV. Analysis included 102 nucleotide sequences (CD-HIT 98%). Relationship was inferred by the maximum likelihood method based on the GTR model with gamma distribution and 1000 bootstrap. Branches were clustered to construct the graph. The first number indicates number of sequences, genotype and subgenotype, and year of detection. Black triangles symbolize clustering of similar sequences identified by number of sequences, genotype and years of report. Colors represent different genotypes, blue for genotype 2, green for genotype 3, magenta for genotype 4, cyan for genotype 5, purple for genotype 6 and light blue for genotype 7.

The second phylogenetic tree was constructed using capsid sequences from the complete genomes, capsid sequence B1 MXCDg3_B1c|_2016 and the partial sequence obtained from the feces samples (MXCDg3_C3Acons|_2016). All sequences cluster with the genotype 3a sequences, as expected (Figure 3).

Afterwards, each ORF was evaluated separately at the nucleotide level with its own phylogenetic analysis. Comparisons showed the same result as for the complete sequence, i.e., clustering with subgenotype 3a [39].

## 4. Discussion

HEV causes apparent asymptomatic infection in swine. However, it is a public health concern as it causes acute hepatitis in humans of varying severity that could lead to chronic affection. Most severe human infections occur in pregnant women and can impact the outcome of the pregnancy (25% mortality rates). HEV is ubiquitous and difficult to detect, and higher seroconversion levels have been documented in people with occupational exposure to swine. This is the first report of a complete genome characterization of hepatitis E virus genotype 3 in Mexico (strain MXCDg3_B1c|_2016). Generating information of the virus is important as it helps establish a reference strain of the virus that circulates in pigs in slaughterhouses and different production stages in the country.

The results of the sampling carried out at four slaughterhouses (64 total samples of liver, bile, and feces) yielded only one positive sample of bile (1.56%). An earlier study conducted in Nuevo Leon, Mexico, had reported HEV sequences in butcher shops with 22.5% positive results; however, we found no positive liver samples at the slaughterhouses [28]. Also, a study conducted in Brazil that analyzed 118 clinically-asymptomatic pigs had found two liver samples (1.7%) and one bile sample (0.84%) that were positive [40]. This result is close to our results, perhaps due to the study design and sampling procedure employed. Sampling represents a limitation due to the lack of a more standardized sampling method; this is especially the case for liver samples where only a small fraction (2 g) of tissue was used for RNA extraction. More information is needed regarding the presence of the virus in the food supply chain, and risk factors should be considered when devising strategies to prevent contamination in the pork food chain.

According to reports by Casas et al., feces sampling (rectal swabs) was carried out on a full-cycle pig farm to assess the zoonotic risk for people who work, or have contact, with pigs—a risk factor that has been reported in several studies [41,42,43,44]. Of the 21 sample pools analyzed, two from the fattening stage were highly positive. These findings coincide with previous reports that identified the elimination peak of the virus in the twelfth week of age [41]. However, some samples from pigs in the gestation, farrowing, and nursing stages were also found to be positive, indicating that the virus circulates in all production stages. These high levels of viral elimination might be related to high anti-HEV antibody prevalence reported previously in farm workers. Among farm samples, 66.66% proved to be positive. This is the first RT-PCR analysis conducted on a farm in Mexico; therefore, no comparison values can be discussed. However, seroprevalence values have been reported. A study conducted on swine farms in different areas of Mexico revealed a HEV IgG seroprevalence of 42.7% in the central area of the country [24]. Another study conducted by Merino Ramos et al. that covered nine central states in Mexico found a 30.75% seroprevalence [25]. Although evidence of the disease exists, the matter has received little interest from authorities.

All necropsy samples were positive, except for one stool sample. It is important to note that all pigs were selected for sacrifice for teaching purposes and were especially selected due to reduced weight gain compared to other pigs in the same stage. HEV has not been directly associated with any clinical manifestations in swine hosts that could affect economic parameters. However, a few specific cases have been reported that showed high morbidity and mortality when HEV was found along with other known pathogenic swine viruses, such as Porcine Circovirus 2 (PCV-2) and porcine reproductive and respiratory syndrome virus (PRRS), both of which are common in Mexican swine farms [45,46]. Further analysis should include evaluation of production parameters during HEV infection to assess the possible effects of the disease in swine populations.

Phylogenetic analysis showed that the virus clusters with genotype 3, subgenotype a (G3a) reported sequences that have been associated with acute hepatitis cases in humans in the US (GenBank AF060669.1) and Japan (GenBank AB089824) as well as in swine from China (GenBank KT727028). The sequences reported in the US (HEV-US1, HEV-US2) were the first identified as genotype 3 due to their low homology with the Burma and Mexico strains (73.5–74.5%). They were isolated from two human patients with acute hepatitis [47]. Genotype 3a was also reported recently in Colombia [48]. It is possible that this is the strain currently circulating in America.

Although HEV has been confirmed as a zoonotic disease, it has received little attention in Mexico, where clinical cases might be underestimated. This is also the situation for other countries in America that are currently defining the importance of this disease [48,49,50,51]. One Health is focused on diseases that generate associations between humans, animals, and the environment; plenty of studies have demonstrated the risk of HEV for humans in association with animals, as a food-borne pathogen, and the risk of environmental transmission. Surveillance of porcine HEV could be helpful in evaluating the potential risk of HEV zoonotic infection in humans [52]. Our findings of HEV in pigs at farm level that shed virus in different stages suggest an occupational risk for swine production workers; therefore, it is necessary to implement biosecurity measures. Some authors have suggested that the difference in age of infection onset or shedding may result from the quantity and/or quality of colostrum that piglets receive with maternal antibodies. In addition, the duration of shedding on farms could be influenced by other pathogens; for instance, one trial showed that PRRSV/HEV coinfection dramatically extended the shedding period by a factor of five [53]. Standard biosecurity measures, including regular cleaning and disinfection, should be implemented to limit fecal contamination of swine facilities. There is scarce information about the pathogenesis of HEV, possible routes of transmission, and vaccine efficacy in swine. Further research should be conducted to address the epidemiology of the disease in farms and develop prevention methods to avoid infection in swine.

The information generated on HEV genotype 3 present in Mexico provides some insight into the disease situation there. Sequences are publicly available and will help develop more specific diagnoses that will provide a broader view of the disease.

## Figures and Tables

**Table 1:** Hepatitis E virus (HEV) PCR results of swine samples from different production stages.

ID	# of Samples	Type of Sample	Origin	Production Stage	RT-PCR Result
1	16	Liver, bile, feces	Slaughterhouse	Finishers	N
2	16	Liver, bile, feces	Slaughterhouse	Finishers	N
3	16	Liver, bile, feces	Slaughterhouse	Finishers	P*
4	16	Liver, bile, feces	Slaughterhouse	Finishers	N
G1	10	Feces	Farm	Gestation	P
G2	10	Feces	Farm	Gestation	N
G3	10	Feces	Farm	Gestation	P
D1	10	Feces	Farm	Weaning	P
D2	10	Feces	Farm	Weaning	P
D3	10	Feces	Farm	Weaning	P
E1	10	Feces	Farm	Finishers	N
E2	10	Feces	Farm	Finishers	P
E12	10	Feces	Farm	Finishers	P
M1	10	Feces	Farm	Nursing	N
M2	10	Feces	Farm	Nursing	P
M3	10	Feces	Farm	Nursing	N
R	10	Feces	Farm	Breeding	N
S	10	Feces	Farm	Boars	N
H1	3	Liver	Farm	Finishers	P
H2	4	Liver	Farm	Finishers	P
H3	1	Liver	Farm	Finishers	P
B1	4	Bile	Farm	Finishers	P
B2	4	Bile	Farm	Finishers	P
C1	4	Feces	Farm	Finishers	P
C2	4	Feces	Farm	Finishers	N
* only 1 bile positive					

P, positive; N, negative; * only 1 bile sample proved to be positive.

**Table 2:** Nucleotide and amino acid identity (%) of swine HEV isolate MXCDg3_B1c|_2016 with complete genome sequences of various genotypes retrieved from GenBank with over 75% homology.

Sequence Identification	Genome	ORF1		ORF2		ORF3	
ID	Full-Length% (nt)	% (nt)	% (aa)	% (nt)	% (aa)	% (nt)	% (aa)
**USHM|AF060669.1|g3a|_1998**	91	90	97	92	98	98	98
**JPHM|AB089824.1|g3|_1993**	91	90	98	92	98	96	98
**USHM|JQ679014.1|g3|_2010**	91	90	97	92	98	96	98
**USCD|AY575857.1|g3|_1997**	91	90	98	92	98	96	98
**USMN|JN837481.1|g3|_2008**	91	90	97	92	99	97	97
**JPHM|AB074920.3|g3|_2002**	91	90	98	92	99	98	98
**JPHM|AB074918.2|g3|_2002**	91	90	98	93	99	98	98
**CNCD|KJ507955.1|g3|_2003**	90	90	98	92	98	97	99
**KRCD|FJ426403.1|g3|_2007**	90	90	96	93	97	96	98
**KRCD|FJ426404.1|g3|_2007**	90	89	96	92	98	97	98
**SGHM|KT447526.1|g3|_2010**	90	89	97	91	98	96	96
**USHM|JQ679013.1|g3|_2010**	90	90	93	92	98	94	97
**CHCD|KX981911.1|g3|_2015**	89	89	96	91	98	97	98
**CNCD|AY115488.1|g3|_2002**	87	86	95	90	98	96	98
**JPHM|AB091394.1|g3|_2002**	87	86	97	89	99	97	97
**JPCD|AB740232.1|g3|_2013**	87	86	96	89	98	96	98
**JPHM|LC131066.1|g3|_2016**	87	86	96	90	99	96	96
**JPHM|AB437317.1|g3|_2003**	87	86	97	89	99	97	96
**JPHM|LC176492.1|g3k|_2015**	87	86	96	90	98	96	96
**JPHM|AB369689.1|g3|_2004**	87	86	96	90	99	97	97
**JPHM|AB291963.1|g3|_2005**	87	86	96	90	98	95	93
**JPJB|AB222184.1|g3|_2004**	87	86	96	88	98	96	96
**JPMN|AB236320.1|g3|_2002**	87	86	97	89	98	97	97
**JPJB|AB222182.1|g3|_2004**	87	86	96	89	98	96	96
**CNCD|KJ507956.1|g3|2003**	87	86	97	89	98	96	93
**JPJB|AB222183.1|g3|_2004**	87	86	97	89	98	96	93
**JPCD|AB073912.1|g3|_2001**	87	86	96	89	98	96	96
**JPJB|AB189070.1|g3|_2004**	87	85	96	89	98	96	96
**JPHM|AB437319.1|g3|2003**	87	91	97	89	99	96	95
**JPHM|AB291960.1|g3|_2006**	86	86	97	89	98	96	95
**JPHM|AB291952.1|g3|_2005**	86	86	97	88	99	97	96
**JPHM|AB369691.1|g3|_2005**	86	86	97	89	99	98	96
**JPHM|AP003430.1|g3|_2001**	86	86	96	88	98	97	98
**JPHM|AB291962.1|g3|_2004**	86	85	96	89	98	96	97
**CHCD|FJ527832.2|g3|2008**	86	86	96	88	98	97	98
**JPND|AB246676.1|g3|_2006**	86	85	97	89	98	99	93
**ALJB|FJ998008.1|g3|_2007**	85	84	96	87	99	95	94
**CHCD|FJ610232.1|G4|_2008**	78	79	85	81	93	87	85
**TWHM|HQ634346.1|G4|_2010**	78	78	86	81	94	90	88
**CHCD|KC692453.1|G4|_2011**	78	78	85	80	94	88	86
**EACM|KJ496143.1|g7|_2013**	78	78	87	80	92	87	80
**CHCD|GU119960.2|G4a|_2009**	78	77	85	81	94	89	88
**JPJB|AB573435.2|G5|_2009**	78	77	84	80	91	86	80
**CDCH|GU206559.1|G4|_2008**	78	79	85	81	92	86	85
**INHM|JF443724.1|G1|_2005**	77	77	82	80	92	85	82
**CHHM|NC_001434.1|G1|_1987**	77	77	82	80	92	85	82
**CHHM|JQ655734.1|G1|_2012**	77	77	82	79	91	86	82
**MXHM|M74506.1|g2|_1992**	76	77	81	79	91	85	80
**JPWB|AB602441.1|g6|_2006**	76	NS	83	79	91	84	73
* NS. No similarity found							
